# Dengue immune sera enhance Zika virus infection in human peripheral blood monocytes through Fc gamma receptors

**DOI:** 10.1371/journal.pone.0200478

**Published:** 2018-07-25

**Authors:** Min Li, Lingzhai Zhao, Chao Zhang, Xin Wang, Wenxin Hong, Jin Sun, Ran Liu, Lei Yu, Jianhua Wang, Fuchun Zhang, Xia Jin

**Affiliations:** 1 CAS Key Laboratory of Molecular Virology and Immunology, Institut Pasteur of Shanghai, Chinese Academy of Sciences (CAS), Shanghai, China; 2 University of Chinese Academy of Sciences, Beijing, China; 3 Guangzhou Eighth People’s Hospital, Guangzhou Medical University, Guangzhou, China; 4 School of Life Sciences, Shanghai University, Shanghai, China; University of Hong Kong, HONG KONG

## Abstract

Antibody dependent enhancement (ADE) has most often been associated with dengue virus (DENV). Studies using leukemia cell lines suggest that DENV specific antibodies can enhance Zika virus (ZIKV) infectivity, and vice versa. To examine the mechanisms of ADE of ZIKV infection in primary human cells, we assessed 40 serum samples obtained from convalescent DENV-1 or DENV-3 infected subjects. All sera tested exhibited high binding potency, while modest or none neutralization activities against ZIKV. Primary CD14+ monocytes, rather than B and T cells in peripheral blood mononuclear cells (PBMCs), were found to be the mediators of the enhancement of ZIKV infectivity by DENV immune sera. Monocyte-derived immature dendritic cells (DCs), but not mature DCs were highly permissive to ZIKV infection, whereas neither immature nor mature DCs could mediate enhanced ZIKV infection in the presence of DENV immune sera. In addition, antibody blocking of either FcγRI (CD64), or FcγRII (CD32), or FcγRIII (CD16) resulted in diminished ADE of ZIKV infection. Our findings provide an improved understanding of the pathogenesis of ZIKV infection, and inform rational vaccine design.

## Introduction

Zika Virus (ZIKV) is a mosquito-borne flavivirus transmitted mostly by *Aedes aegypti*. It has evolved into two distinct genetic lineages: African and Asian types [1]. Since its first isolation from a captive rhesus monkey in the Zika forest of Uganda in 1947, ZIKV had transmitted in obscurity for decades during the 20th century with sporadic human cases that manifested with mild symptoms such as fever, rash, headache, conjunctivitis, arthralgia and myalgia [2, 3]. In 2007, ZIKV produced its first noted outbreak on Yap Island, and then sparked a subsequent outbreak in French Polynesia during 2013–2014, followed by a major epidemic during 2015–2016 in Brazil and more than 80 other countries across five continents that accrued more than 2 million reported cases, coupled with dramatic increased incidence of Guillain-Barré syndrome in adults and microcephaly in the developing fetuses [4–8]. The exact reasons for the ZIKV pandemics are unknown, but preexisting DENV immunity was quite common (over 80%) among ZIKV infected patients in the 2013–2014 French Polynesia outbreak [5]. Similarly, another ZIKV endemic region, Brazil, also experienced widespread DENV disseminations including all four DENV serotypes in recent years [9]. Such unprecedented ZIKV outbreaks and co-circulation of genetically and structurally closely associated DENV drew intense investigations into the interactions between these two viruses.

In mouse and nonhuman primate models, as well as anecdotal human case studies, certain features of ZIKV tropism have been revealed. ZIKV may damage brain tissue via infection of human neural progenitor cells, providing a plausible explanation for the cause of microcephaly; human placental cells are permissive to ZIKV infection *in vitro*, suggesting the route for vertical transmission [10–14]; additionally, persistent infectious virus or viral RNA could be detected in a variety of body fluids including tears, saliva, urine, semen [15–17], offering a mechanism for sexual transmission. Meanwhile, the early target cells in human blood circulatory system might have helped the spread and amplification of the virus soon after its percutaneous inoculation. Studies elucidated that human CD14+ monocytes and monocyte-derived dendritic cells (moDC) from human peripheral mononuclear cells (PBMCs) facilitated ZIKV replication [18–20]. Whether these target cells in human blood can mediate antibody-dependent enhancement (ADE) of ZIKV infection is yet to be determined.

Host cells that display putative ZIKV receptors may be susceptible to ZIKV infection, and cells bearing Fcγ receptors (FcγRs) may facilitate virus infection through binding and internalizing infectious virus-antibody complexes as well. The ADE phenomenon has long been recognized during DENV infection *in vitro* and *in vivo* [21–23]. In experimental models, it is clear now that anti-DENV antibodies can mediate ADE of ZIKV infection, and vice versa [24–27], possibly due to the sequence and structural similarity between DENV and ZIKV [28, 29]. Notably, the envelope (E) proteins of ZIKV and DENV share high sequence identity (over 50%) [25, 28], making these antigenic sites prone to induce cross-reactive antibodies between DENV and ZIKV.

In the current study, we provide evidence for the first time that human primary monocytes, rather than B cells, T cells and dendritic cells (DCs) are principal mediators of ADE infection of ZIKV by DENV immune sera. Moreover, times post-infection, instead of DENV serotype distinction, potentially affect the magnitude of serological cross-reactivity between DENV immune sera and ZIKV, and the capacity of these sera to enhance ZIKV infection *in vitro*. Furthermore, all three types of FcγRs expressed on human primary monocytes contribute to enhanced ZIKV infection in the presence of DENV immune human sera. These results provide more insight into the pathogenesis of ZIKV infection, and the complex interplay among flaviviruses.

## Materials and methods

### Human samples

DENV immune serum samples were collected by physicians at Guangzhou Eighth People’s Hospital (Guangzhou, China) from convalescent patients who were infected by either DENV-1 or DENV-3 in Guangdong province (DENV-1, China) in 2014 or Yunnan province (DENV-3, China) in 2013. DENV-1 immune serum samples were collected between September 2014 and March 2015, and DENV-3 immune serum samples were collected between October 2013 and March 2014. The serotype of DENV infection (during acute phase) was determined by real-time polymerase chain reaction (RT-PCR) detection of viral genome using serotype specific primers. The primary and secondary DENV infections were determined by anti-DENV IgG level and anti-DENV IgM/IgG ratio according to the published method [30]. Written informed consent was obtained from each of the research participants. The study was conducted from January 2017, and was approved by the Ethics Committee of the Institut Pasteur of Shanghai (Number: IPS-2016009) and that of the Guangzhou Eighth People’s Hospital (Number: 20131224) in China.

### Cells and viruses

Vero cells were grown in Dulbecco’s Modified Eagle Medium (DMEM, Gibco) supplemented with 10% heat-inactivated fetal bovine serum (Gibco) at 37°C with 5% CO_2_. C6/36 *Aedes albopictus* cells were maintained in Minimum Essential Medium (MEM, Gibco) supplemented with 10% heat-inactivated fetal bovine serum and non-essential amino acids (Gibco) at 28°C with 5% CO_2_. All medium contained 50 U/ml penicillin (Gibco) and 50 μg/ml streptomycin (Gibco). The Asian ZIKV strain SZ-WIV01, isolated from the serum of an imported ZIKV-infected case in China in 2016, was obtained from Wuhan Institute of Virology (Chinese Academy of Sciences). DENV-1 (strain 16007) and DENV-3 (strain 16562) were gifts from Dr. Claire Huang (U.S. Centers for Disease Control and Prevention at Fort Collins, Colorado). All viruses were grown in C6/36 *Aedes albopictus* cells and titrated on Vero cells by a plaque-forming assay (ZIKV) or a focus-forming assay (DENV).

### Primary cell isolation

Peripheral blood mononuclear cells (PBMCs) used were isolated by standard density centrifugation procedures with Ficoll-Paque PLUS (GE Healthcare) from fresh buffy coats of healthy donors collected by licensed physicians at the Shanghai Blood Center (Shanghai, China). PBMCs were used immediately after isolation. CD14 positive and negative populations were fractionated from PBMCs using magnetic microbeads conjugated with anti-human CD14 antibodies (Miltenyi Biotec) following the manufacturer’s instruction. For generation of monocyte-derived dendritic cells, CD14 positive monocytes were resuspended in Roswell Park Memorial Institute 1640 (RPMI-1640) medium supplemented with 10% heat-inactivated fetal bovine serum, 50 U/ml penicillin, 50 μg/ml streptomycin, 2 mM of L-glutamine (Gibco), 1 mM N-2-hydroxyethylpiperazine-N-2-ethane sulfonic acid (HEPES, Gibco) and 100 ng/ml each of recombinant human interleukin-4 (IL-4, Peprotech) and granulocyte-macrophage colony stimulating factor (GM-CSF, PeproTech), and incubated for 6 days at 37°C with 5% CO_2_. On day 5, cell maturation was induced by stimulation with 100 ng/ml Lipopolysaccharide (LPS, Sigma) for 2 days. Immature DCs were characterized as CD14 low/-, HLA-DR+, CD11c+, DC-SIGN+, CD83- and CD86 low cells by flow cytometry. Mature DCs express additionally CD83 and high levels of CD86.

### Measurement of ZIKV binding capacity with ELISA

High-binding 96 well plates (Corning, New York) were pre-coated with 10 μg/ml concanavalin A (Sigma), a plant lectin that binds to glycoproteins [31]. Wells were washed and then incubated with UV-inactivated ZIKV (1×10^5^ plaque forming unit (PFU) /well) overnight at 4°C. After blocking with 5% bovine serum albumin (Sigma) in PBS containing 0.05% Tween-20, serially diluted inactivated serum samples were added into plates for 1 h at room temperature. Reactions were visualized by incubation with horseradish peroxidase (HRP)-conjugated anti-human IgG antibody (Abcam), followed by adding tetramethylbenzidine (TMB) substrate (Life Technologies). The endpoint titer was defined as the reciprocal of the lowest serum dilution above two times the average OD450 values of wells without applying serum samples.

### Neutralization assay

Serotype-specific and ZIKV cross-reactive neutralization activities of sera from DENV-infected patients were detected by performing a focus-forming assay (DENV) or a plaque-forming assay (ZIKV) on Vero cells. Specifically, heat-inactivated serum samples were two-fold serially diluted and incubated with DENV (80–100 focus forming unit (FFU)) or ZIKV (50–70 PFU) for 1 h at 37°C before applied to Vero cells. Subsequently, antibody-virus mixture was aspirated from cells, and an overlay of 1% carboxyl methyl cellulose (Sigma) in DMEM containing 1.5% heat-inactivated fetal bovine serum was applied to each well. After a 3- or 4-day incubation at 37°C, cells were washed with PBS and fixed with 4% paraformaldehyde (PFA). For DENV-1 and DENV-3 infection, foci were stained using DENV E protein specific antibody D1-11 (Santa Cruz Biotechnology) for 2 h, followed by incubation with biotin-conjugated anti-mouse IgG antibody (Santa Cruz Biotechnology) for another 2 h and a subsequent incubation with streptavidin-alkaline phosphatase (streptavidin-AP, Sigma) for 2 h, and finally developed with 5-bromo-4-chloro-3-indolyl-phosphate/nitro blue tetrazolium substrate (Beyotime Technology). For ZIKV infection, plaques were visualized by staining with 0.5% crystal violet and counted manually. The 50% plaque reduction neutralization titer (PRNT_50_) was calculated by probit analysis using the SPSS software.

### Antibody-dependent enhancement of ZIKV infection in primary cells

Heat-inactivated human serum samples were five-fold serially diluted with RPMI-1640 (Gibco), and mixed with ZIKV at a multiplicity of infection (MOI) of 2 for 1 h at 37°C, then added to freshly isolated human PBMCs from healthy donors or monocyte-derived DCs (immature/mature). After incubation for 1.5 h at 37°C, cells were then washed twice, resuspended in RPMI-1640 medium supplemented with 10% heat-inactivated fetal bovine serum, 50 U/ml penicillin, 50 μg/ml streptomycin and 2 mM of L-glutamine (Gibco), and cultured at 37°C for an additional 2 days. DENV immune sera mediated antibody-dependent enhancement of ZIKV infection was then evaluated by cell staining and subsequent flow cytometry analyses. Infected cells were harvested and stained intracellularly for ZIKV antigen detection using a pan-flavivirus specific antibody (4G2), and additionally surface stained with a panel of cell surface markers to identify specific cell subsets for PBMCs. The percentage of ZIKV infected cells of a unique cell subset (CD3+CD19-CD14- T cells, CD3-CD19+CD14- B cells, CD3-CD19-CD14+ monocytes) within PBMCs or intracellularly stained DCs was analyzed by flow cytometry.

### Boosted infection assays on Vero cells

To amplify the weak signals of anti-E positive staining in ADE assays using primary cells and to demonstrate the small proportion of anti-E positive cells generated infectious progenies, we performed boosted infection assays on Vero cells that are highly sensitive to ZIKV infection. A total of 200 μl cell culture supernatant from infected PBMCs or DCs was inoculated into Vero cells. After incubation at 37°C for 1 h, Vero cells were subsequently washed once and cultured with DMEM containing 2% heat-inactivated fetal bovine serum for ZIKV amplification for another 2 days before being applied to detect ZIKV infectivity by flow cytometry. Fold enhancement was analyzed by comparison of the percentage of infected cells in the presence of serum samples to that in the absence of serum samples.

### Flow cytometry analysis

Mouse anti-human antibodies anti-CD3 (PE-Cy7; clone UCHT1; Biolegend), anti-CD4 (BV-785; clone RPA-T4; Biolegend), anti-CD14 (PE; clone M5E2; Biolegend), anti-CD19 (BV-711; clone HIB19; Biolegend), anti-CD19 (APC; clone HIB19; Biolegend), anti-HLA-DR (Percp; clone L243; Biolegend), anti-CD11c (APC; clone 3.9; eBioscience), anti-DC-SIGN (PE-Cy7; clone 9E9A8; Biolegend), anti-CD83 (FITC; clone HB15e; Biolegend) and anti-CD86 (PE; clone IT2.2; eBioscience) were purchased from Biolegend and eBioscience. Isotype control murine immunoglobulin IgG1 κ (FITC; APC; clone P3.6.2.8.1), mouse IgG2a κ (PE; PE-Cy7; Alexa Fluor 488; clone eBM2a), mouse IgG2b κ (PE; clone eBMG2b) were purchased from eBioscience. For myeloid dendritic cell (mDC) staining within PBMCs and monocyte-derived dendritic cell characterization, freshly isolated PBMCs and DCs were blocked with either 10% fetal bovine serum (PBMCs) or 10% normal human serum (DCs) for 30 min at 4°C, followed by incubation with surface markers and viability staining (aqua fluorescent reactive dye, Life Technologies) for 30 min at 4°C in the dark. For ZIKV- infected PBMCs or DCs, cells were washed once with PBS, then immediately stained with aqua fluorescent reactive dye (Life Technologies) to evaluate the viability of cells. Subsequently, cells were fixed and permeabilized with fixation/permeabilization solution (BD Biosciences) followed by intracellularly stained for 30 min at 4°C using a pan-flavivirus antibody 4G2 that recognized the fusion loop of E protein. After incubation with anti-mouse IgG Alexa Fluor 488 (Life Technologies) for another 30 min at 4°C, PBMCs were washed thoroughly and applied for cell surface staining with anti-CD3, anti-CD4, anti-CD14 and anti-CD19 antibodies for 30 min at 4°C in the dark. For ZIKV-infected Vero cells, cell monolayers were washed once with PBS followed by digestion with trypsin-EDTA (Gibco). Cells were fixed and permeabilized, further stained with an anti-E antibody (4G2) and a secondary anti-mouse IgG antibody (Alexa Fluor 488) for flow cytometry analysis. Data acquisition was processed on a LSR Fortessa (BD Biosciences) or a LSRII (BD Biosciences) cytometer and the acquired data were analyzed using FlowJo software (TriStar). The minimum events recorded were 4×10^5^, 7×10^4^, 1.2×10^5^, 1×10^5^ for ZIKV infected PBMCs, immature DCs, mature DCs and Vero cells, respectively.

### FcγR blocking using monoclonal antibodies

Freshly isolated PBMCs from healthy donors were first incubated with anti-CD16 (clone 3G8; Biolegend) or anti-CD32 (clone IV.3; GeneTex) or anti-CD64 (clone 10.1; GeneTex), respectively, at a range of antibody concentrations (1, 3, 10 μg/ml) or three antibodies combined (1, 3 or 10 μg/ml in total, equal amount of each antibody) for 1 h at 4°C. Mouse IgG isotype (GeneTex) was utilized as a negative control in the same range of concentrations (1, 3, 10 μg/ml). After incubation, cells were washed twice with RPMI-1640 medium followed by infection with ZIKV only (MOI 2) or ZIKV that had been pre-incubated with DENV immune serum complexes (MOI 2, D1-E01 serum sample at the dilution of 1/2,500) for 1.5 h at 37°C. Cells were subsequently washed once and cultured in RPMI-1640 medium containing 10% heat-inactivated fetal bovine serum, 50 U/ml penicillin, 50 μg/ml streptomycin and 2 mM of L-glutamine at 37°C for 2 days. Following 2-day culture, 200 μl cell supernatants were harvested for further ZIKV amplification in Vero cells for an additional 2 days. Infected Vero cells were intracellularly stained with 4G2 antibody and the proportion of ZIKV infected cells was measured by flow cytometry.

### Statistical analyses

For virus neutralization assay, the 50% plaque reduction neutralization titer (PRNT_50_) was calculated by probit analysis using SPSS Statistics version 22 (IBM). Statistical significance was determined either by two-tailed unpaired Student’s *t* test or Mann-Whitney test using Graphpad Prism 6.0 software. Analyzes with a P value < 0.05 were considered statistically significant.

## Results

### Convalescent DENV immune human sera are cross-reactive with ZIKV

**Fig 1:**
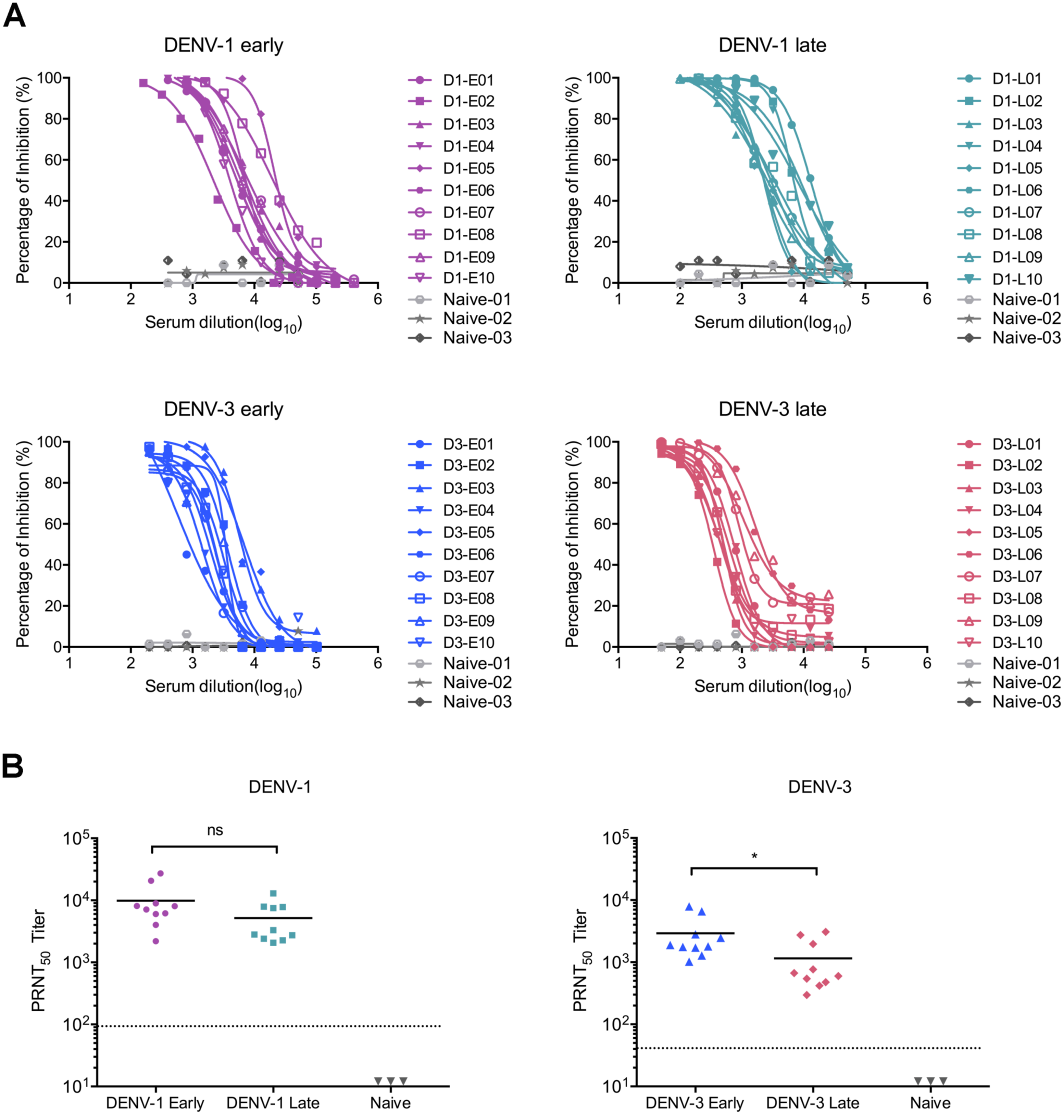
Serotype-specific neutralization activities of serum samples from convalescent dengue patients. (A) Serotype-specific neutralization potency against DENV-1 or DENV-3 was determined by performing focus-forming assays on Vero cells with 2-fold serially diluted DENV-naïve sera (n = 3) and DENV immune sera of DENV-1 early (n = 10), DENV-1 late (n = 10), DENV-3 early (n = 10) and DENV-3 late (n = 10) groups, respectively. (B) The 50% plaque reduction neutralization end-point titers (PRNT_50_) against either DENV-1 or DENV-3 were calculated by probit analysis using the SPSS software. Small horizontal solid lines indicated the mean values of PRNT_50_, and the dotted lines represented the initial serum dilution (1/100 for DENV-1 and 1/50 for DENV-3). Statistical significances were identified using unpaired two-tailed Student’s *t* test. “*” represents p<0.05.

To elucidate the potential role of preexisting DENV immune responses on ZIKV infectivity, a total of 40 convalescent human serum samples from 20 DENV-1 and 20 DENV-3 infected individuals were included in this study, with approximately equal distributions of gender and primary/secondary infection status as determined by the anti-DENV IgM/IgG responses (Table 1). All the samples were collected from infected individuals in Guangdong province (DENV-1) or Yunnan province (DENV-3) in China during 2013–2015, when there were large DENV outbreaks in China, and at a time when no ZIKV cases had been reported in China (The first imported case of ZIKV infection in China was confirmed in February 2016). Sera were collected from 19 day to 212 day following the onset of clinical symptoms for DENV-1 or DENV-3 infection. Based on different sampling times following the onset of disease, DENV immune sera were divided into four different groups: DENV-1 early (19 day to 79 day, n = 10), DENV-1 late (153 day to 199 day, n = 10), DENV-3 early (24 day to 64 day, n = 10) and DENV-3 late (173 day to 212 day, n = 10). In all cases, DENV immune sera presented strong serotype-specific neutralization potency, with similar PRNT_50_ values in early and late sampling groups of DENV-1 immune sera, and a higher average PRNT_50_ value in DENV-3 early group than that of the late group (Fig 1 and Table 1). Additionally, serum samples from three DENV-naïve healthy donors were also included as negative controls (Table 1).

**Table 1:** Characteristics of study subjects.

Patient ID	Age (years)	Sex	Infection serotype	Primary or secondary Infection	Onset of disease	PRNT_50_*		
						DENV-1	DENV-3	ZIKV
D1-E01	73	Male	DENV-1	Secondary	20 day	8128	N.A.^§^	<10
D1-E02	42	Male	DENV-1	Primary	79 day	2196	N.A.	<10
D1-E03	60	Male	DENV-1	Secondary	29 day	8054	N.A.	<10
D1-E04	71	Female	DENV-1	Secondary	46 day	8913	N.A.	<10
D1-E05	84	Female	DENV-1	Secondary	27 day	27102	N.A.	515
D1-E06	98	Female	DENV-1	Secondary	19 day	6180	N.A.	<10
D1-E07	27	Male	DENV-1	Primary	22 day	5998	N.A.	<10
D1-E08	42	Female	DENV-1	Primary	19 day	20654	N.A.	<10
D1-E09	73	Male	DENV-1	Primary	23 day	7112	N.A.	<10
D1-E10	73	Male	DENV-1	Primary	24 day	4009	N.A.	11
D1-L01	51	Female	DENV-1	Primary	165 day	12912	N.A.	<10
D1-L02	43	Male	DENV-1	Primary	171 day	7889	N.A.	12
D1-L03	65	Male	DENV-1	N.A.	189 day	2812	N.A.	12
D1-L04	36	Male	DENV-1	N.A.	153 day	7516	N.A.	<10
D1-L05	47	Male	DENV-1	N.A.	181 day	2075	N.A.	-^¶^
D1-L06	56	Male	DENV-1	Primary	160 day	2410	N.A.	<10
D1-L07	75	Male	DENV-1	Secondary	199 day	2735	N.A.	<10
D1-L08	40	Female	DENV-1	Primary	193 day	3304	N.A.	<10
D1-L09	53	Male	DENV-1	Secondary	173 day	2275	N.A.	13
D1-L10	67	Female	DENV-1	Secondary	171 day	7780	N.A.	<10
D3-E01	45	Female	DENV-3	N.A.	61 day	N.A.	1019	<10
D3-E02	78	Female	DENV-3	N.A.	27 day	N.A.	2805	-
D3-E03	29	Male	DENV-3	Secondary	35 day	N.A.	7889	<10
D3-E04	32	Female	DENV-3	N.A.	59 day	N.A.	1282	-
D3-E05	45	Female	DENV-3	Primary	32 day	N.A.	6576	<10
D3-E06	28	Female	DENV-3	Primary	24 day	N.A.	2471	<10
D3-E07	40	Male	DENV-3	N.A.	58 day	N.A.	1718	<10
D3-E08	22	Female	DENV-3	Primary	25 day	N.A.	1791	12
D3-E09	27	Male	DENV-3	N.A.	64 day	N.A.	1892	<10
D3-E10	41	Male	DENV-3	Secondary	54 day	N.A.	1762	<10
D3-L01	18	Male	DENV-3	Secondary	212 day	N.A.	667	<10
D3-L02	20	Male	DENV-3	Secondary	191 day	N.A.	296	<10
D3-L03	34	Female	DENV-3	Primary	184 day	N.A.	478	<10
D3-L04	22	Female	DENV-3	Primary	178 day	N.A.	597	<10
D3-L05	72	Male	DENV-3	Primary	203 day	N.A.	418	-
D3-L06	49	Female	DENV-3	Secondary	192 day	N.A.	3076	<10
D3-L07	18	Male	DENV-3	Primary	173 day	N.A.	1963	<10
D3-L08	42	Male	DENV-3	Secondary	182 day	N.A.	764	-
D3-L09	16	Female	DENV-3	Primary	208 day	N.A.	2735	<10
D3-L10	44	Female	DENV-3	Secondary	185 day	N.A.	542	<10
Naïve-01	26	Male	/	/	/	-	-	-
Naïve-02	33	Female	/	/	/	-	-	-
Naïve-03	25	Male	/	/	/	-	-	-

* The 50% plaque reduction neutralization titer (PRNT50) was calculated by probit analysis using SPSS Statistics version 22 (IBM);

^§^ N.A.: not applicable;

^¶^ “-” indicates no neutralization activity was observed;

**Fig 2:**
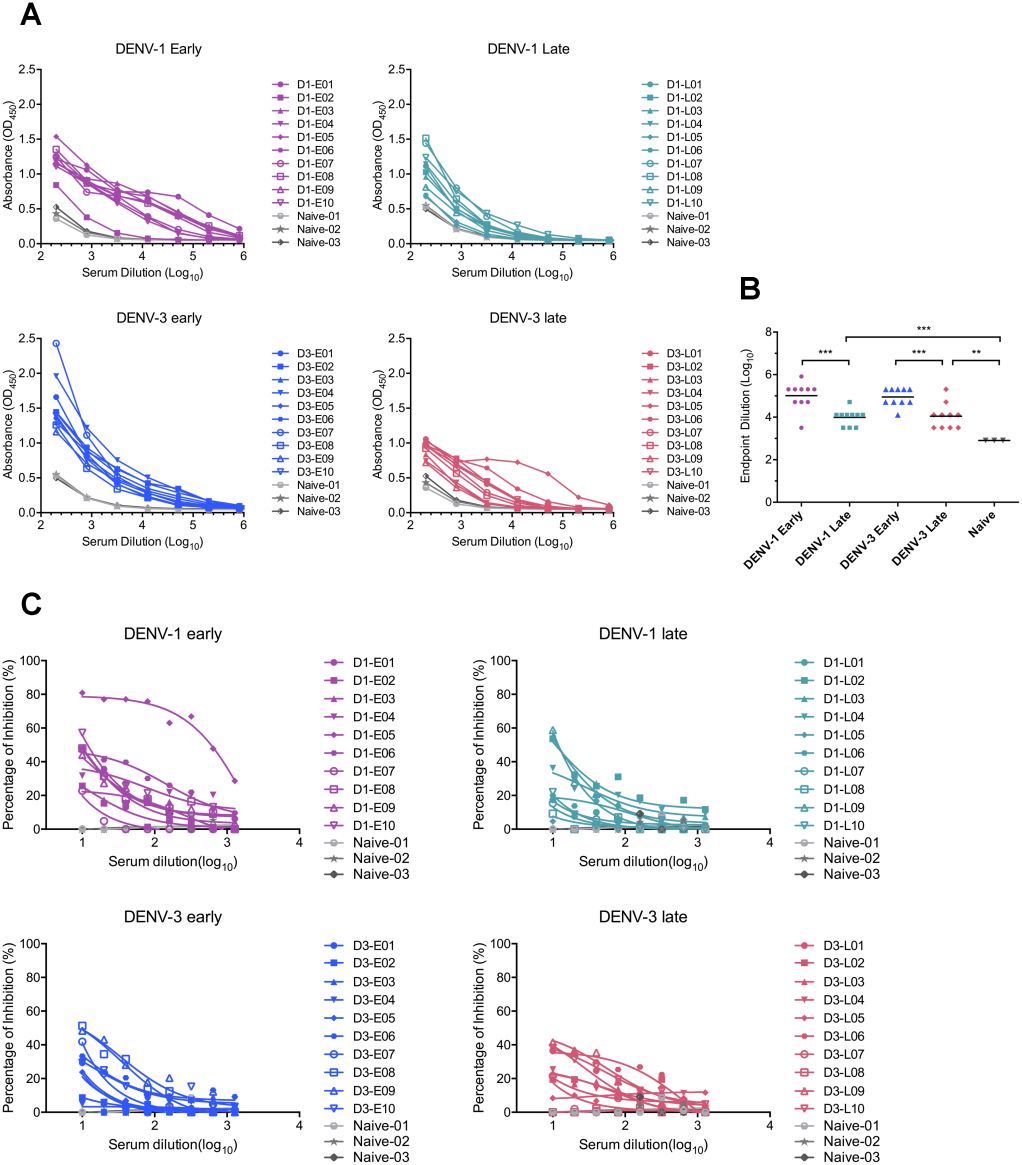
ZIKV binding and neutralization potency by DENV immune sera. (A) Binding capacity of DENV immune sera in DENV-1 early group (n = 10), DENV-1 late group (n = 10), DENV-3 early group (n = 10), DENV-3 late group (n = 10) or DENV-naïve donors (n = 3) were detected by ELISA using plates coated with ZIKV culture supernatant. The binding activity was presented as the optical density value at 450nm (OD450). (B) Summary of ELISA results as mean endpoint titers. Statistical significances were identified using the unpaired two-tailed Student’s *t* test. “**” represents p<0.01, and “***” represents p<0.001. (C) Neutralization potency of DENV-immune sera in each group and three DENV-naïve sera against ZIKV was assessed by performing a plaque-forming assay on Vero cells with 2-fold diluted serum samples.

Considering the high degree of similarities in amino acid sequence and virion structure between ZIKV and DENV, we first determined the binding capacity of DENV immune sera to ZIKV by performing an ELISA. Serial diluted serum samples were applied to the plate pre-coated with ZIKV from culture supernatants, followed by detection with an anti-human IgG secondary antibody and subsequent TMB substrate. Compared with DENV-naïve serum samples, both DENV-1 and DENV-3 immune sera recognized ZIKV efficiently, regardless of age or gender or immune status (primary/secondary infection), with mean endpoint dilution titers of 102,400; 9,701; 89,144; 11,143 for DENV-1 early, DENV-1 late, DENV-3 early and DENV-3 late group, respectively (Fig 2A and 2B). Notably, samples collected from early convalescent phase manifested markedly higher binding activity to ZIKV than that of samples obtained in late sampling times, regardless of DENV serotypes. These results suggest that sampling times of convalescent sera from DENV immune individuals may impact the cross-reactive antibody titers towards ZIKV.

### Convalescent DENV immune human sera exhibit limited neutralization activity against ZIKV

Because DENV-immune sera presented high cross-reactive activity to ZIKV, we subsequently evaluated their neutralization potency against ZIKV infection. Plaque reduction neutralization analysis on Vero cells revealed that the majority of convalescent serum samples collected from DENV-infected subjects presented low to medium neutralization activities against ZIKV (Fig 2C). The most potent neutralizer was a sample obtained from DENV-1 infected individual D1-E05 with a PRNT_50_ titer of 515. Overall, 5/20 of DENV-1 immune sera and 1/20 DENV-3 immune samples showed modest (PRNT_50_ >10) neutralization activity, while remaining samples neutralized ZIKV poorly (PRNT_50_ <10), but one DENV-1 immune sample and 4 samples in DENV-3 immune group showed no appreciable neutralization activity (Table 1).

### Primary monocytes mediate enhanced ZIKV infection in the presence of DENV immune sera

**Fig 3:**
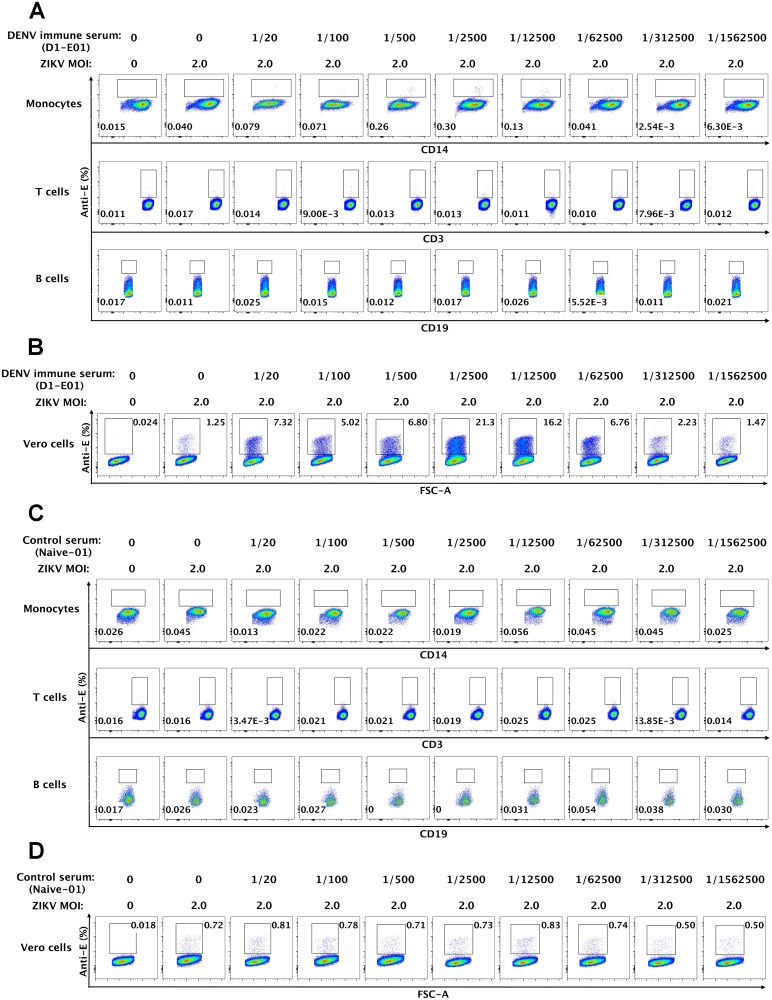
Enhancement of ZIKV infection in primary monocytes triggered by DENV immune sera. Freshly isolated PBMCs from healthy donors were either uninfected or infected with ZIKV at an MOI of 2 in the presence or absence of serially diluted DENV immune serum samples. Cells were harvested and stained with anti-E antibody in combination with antibodies to CD3, CD14, CD19 and then analyzed by flow cytometry. (A) and (C) Representative flow cytometry analyses of ZIKV-infected (Anti-E) monocytes (CD14+CD3-CD19-), T cells (CD14-CD3+CD19-) and B cells (CD14-CD3-CD19+) in the presence of a DENV immune serum sample D1-E01 (A) and a control serum Naïve-01 (C) were presented. (B) and (D) Verification of enhancement in ZIKV infection by the boosted assay on Vero cells. Culture supernatants from ZIKV-infected PBMCs were harvested and used to infect Vero cells for two days followed by intracellular staining with an anti-E antibody and flow cytometry analysis. Representative results of the boosted infection assay on Vero cells for the DENV immune serum D1-01 (B) and the control serum Naïve-01 (D) were shown.

Having characterized the binding and neutralization activity of DENV immune sera against ZIKV, we went on to examine whether these serum samples could mediate ADE infection of ZIKV. We and others have previously reported divergent roles of primary mononuclear phagocytes, B cells and T cells in enhancement of DENV infection [32–35]. Similar methodologies were applied to investigate the relative ZIKV permissiveness among multiple cell subsets in human peripheral blood with or without the presence of DENV immune sera. Freshly isolated PBMCs from healthy donors were inoculated with ZIKV at an MOI of 2 in the presence or absence of serial diluted DENV immune serum samples. ZIKV infectivity of monocytes, T and B cells was assessed at two days post-infection, with intracellularly staining by an anti-E antibody in combination of cell surface staining with CD14, CD3 and CD19 antibodies that identify monocytes, T cells and B cells, respectively (S1 Fig). As a representative result shown in Fig 3A using a DENV-1 immune serum sample (D1-E01), among major cell types of interest, we observed significant elevated ZIKV infection in monocytes (CD14+CD3-CD19-) over a range of serum dilutions (1/20-1/12,500), with the peak enhancement (0.30% infection) at the dilution rate of 1/2,500 compared to the percentage of ZIKV infected cells without serum treatment (0.040% infection). In comparison, the proportions of anti-E positive T cells (CD3+CD14-CD19-) or B cells (CD19+CD3-CD14-) in the presence of serum approximated those in controls, with or without the addition of ZIKV (Fig 3A and Table 2). Accordingly, sera from healthy donors presented no enhancing activities to ZIKV infection in either monocytes or T cells or B cells within PBMCs. (Fig 3C and Table 2). Intriguingly, there appeared to be low but significant levels of ZIKV infection in CD14+ monocytes even in the absence of enhancing antibodies (naïve versus infected monocytes: 0.018±0.010 versus 0.040±0.025, P = 0.012, Table 2), suggesting these may be the target cells of natural ZIKV infection.

**Table 2:** ZIKV-susceptibility in subsets of human PBMCs.

Treatment		^b^Percentage of ZIKV-infected cells (%)		
^a^MOI	Human sera	T cells	B cells	Monocytes
0	0	0.011±0.007	0.042±0.031	0.018±0.010
2	0	0.012±0.006	0.050±0.027	0.040±0.025^#^
**DENV-naïve sera (n = 3)**				
2	1/20	0.011±0.001	0.035±0.021	0.032±0.035
2	1/100	0.010±0.004	0.046±0.022	0.034±0.019
2	1/500	0.015±0.011	0.048±0.041	0.041±0.038
2	1/2500	0.011±0.003	0.063±0.025	0.052±0.020
2	1/12500	0.012±0.001	0.057±0.034	0.050±0.056
2	1/62500	0.012±0.006	0.054±0.049	0.054±0.055
2	1/312500	0.015±0.004	0.035±0.012	0.045±0.039
2	1/1562500	0.043±0.049	0.046±0.020	0.047±0.006
**DENV-1 immune sera (n = 20)**				
2	1/20	0.015±0.016	0.061±0.054	0.257±0.230**
2	1/100	0.011±0.005	0.052±0.033	0.135±0.107*
2	1/500	0.018±0.021	0.060±0.041	0.136±0.111
2	1/2500	0.015±0.013	0.064±0.039	0.105±0.094
2	1/12500	0.014±0.010	0.088±0.059	0.051±0.039
2	1/62500	0.013±0.007	0.062±0.049	0.041±0.031
2	1/312500	0.010±0.005	0.059±0.043	0.039±0.038
2	1/1562500	0.010±0.006	0.067±0.051	0.031±0.020
**DENV-3 immune sera (n = 20)**				
2	1/20	0.015±0.007	0.052±0.045	0.206±0.165*
2	1/100	0.015±0.016	0.049±0.038	0.178±0.178*
2	1/500	0.013±0.004	0.062±0.070	0.116±0.121
2	1/2500	0.008±0.007	0.051±0.056	0.066±0.087
2	1/12500	0.011±0.004	0.065±0.065	0.046±0.039
2	1/62500	0.012±0.010	0.065±0.063	0.037±0.034
2	1/312500	0.012±0.008	0.054±0.042	0.035±0.030
2	1/1562500	0.014±0.023	0.061±0.082	0.033±0.032

^a^ MOI, multiplicity of infection;

^b^ Mean± SD is shown;

“*”, “**” symbols represent significance levers of either P<0.05 or P<0.01 (Mann-Whitney test), in comparison to the percentage of anti-E staining on ZIKV infected cells in the presence of DENV-naïve sera under the same treatment.

^#^ Statistical significance between ZIKV infected monocytes and uninfected monocytes was determined using the Mann-Whitney test. Naïve versus infected monocytes: 0.018±0.010 versus 0.040±0.025, P = 0.012.

To prove that the small proportion of anti-E positive staining in CD14+ monocytes was not an artifact of non-specific antibody staining, but a genuine reflection of ZIKV-infection and produced infectious progeny, we performed a boosted infection assay with Vero cells that are highly permissive to ZIKV. Supernatants from each of ZIKV-inoculated PBMCs in the presence or absence of serum samples were harvested and immediately inoculated onto Vero cells. The extent of ZIKV infection of Vero cells was determined by intracellularly staining with anti-E specific antibody followed by flow cytometry analysis. A similar pattern but a greater magnitude of infection and enhanced ZIKV infection were more evident (Fig 3B). As expected, the DENV immune serum sample (D1-E01) markedly increased ZIKV infection at a wide range of dilutions (1/20-1/62,500) with an approximately 17-fold peak enhancement in the presence of a 1/2,500 dilution of serum. Even in the absence of the DENV immune serum, 1.25% of infected cells were observed (Fig 3B). With a significant increase in the sensitivity of detection of viral infection, the boosted-assay did not increase background staining appreciably. In the negative control group in which no virus was added, there was only 0.024% cells stained positive. In comparison, the proportions of ZIKV infected Vero cells in the boosted infection assay using supernatants from ZIKV infected PBMCs in the presence of control serum were similar to that of infected cells in the absence of the serum sample (Fig 3D).

**Fig 4:**
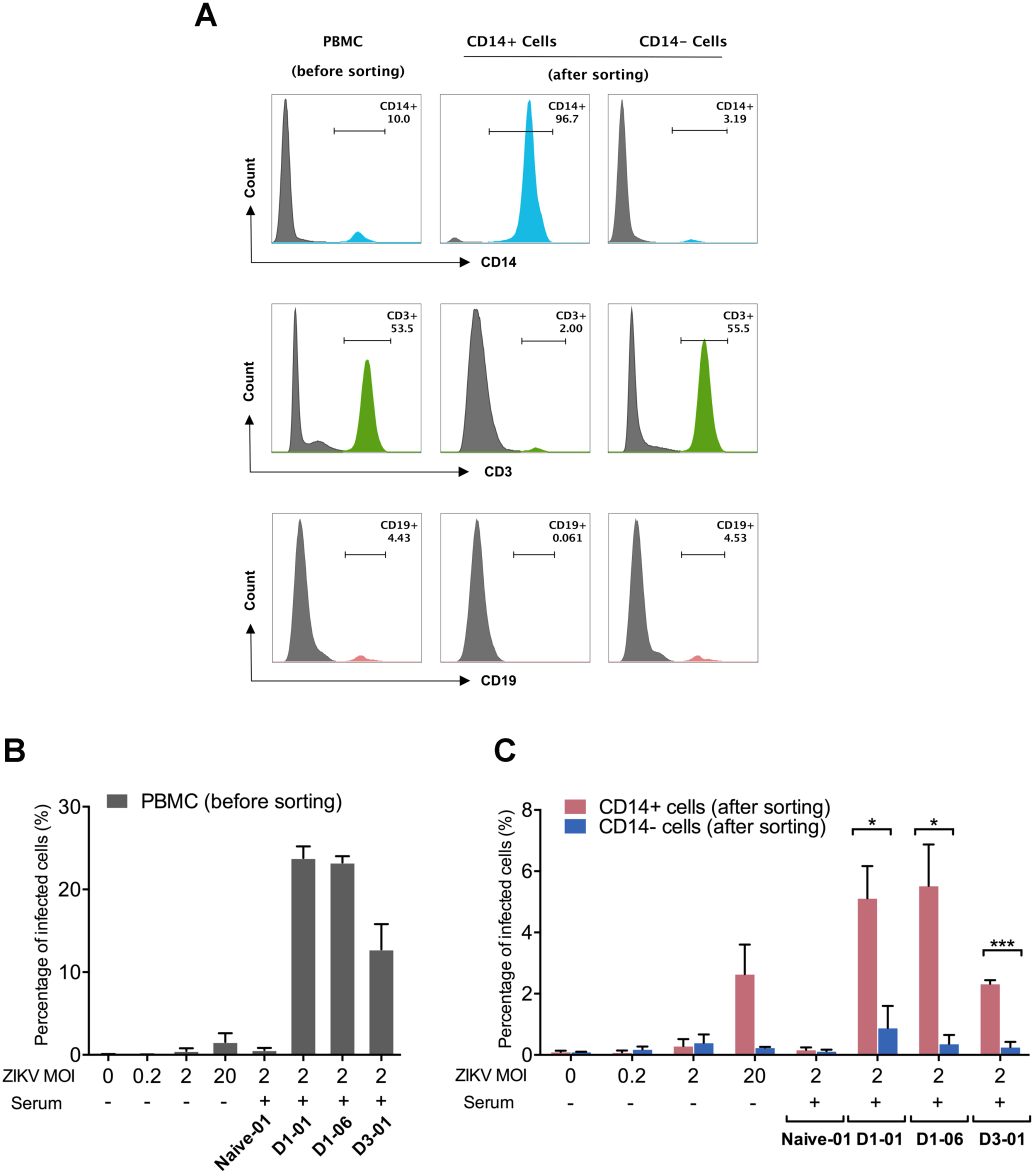
CD14+ monocytes are the principle target cells mediating ADE of ZIKV infection by DENV immune sera. (A) PBMCs were sorted by magnetic beads into CD14+ and CD14- fractions through a positive selection process. The percentage of different cell subtypes (viable cells) in each fraction and unfractionated PBMCs were presented. (B and C) PBMCs as well as CD14+ and CD14- fractions were infected with ZIKV at varying MOIs in the absence of serum samples or an MOI of 2 in the presence of DENV immune sera (D1-E01 at a dilution of 1/2,500, or D1-E06 at a dilution of 1/2,500, or D3-L01 at a dilution of 1/100) or a control serum (Naïve-01, 1/100 dilution). Cell culture supernatants from infected PBMCs (B), CD14+ and CD14- fractions (C) were collected and inoculated on Vero cells and analyzed for ZIKV-infected cells by flow cytometry. The mean and SEM of results from three independent experiments were presented. Statistical significances were determined using unpaired two-tailed Student’s *t* test. “*” represents p<0.05, and “***” represents p<0.001.

To more directly validate that primary human peripheral monocytes were the principal target cells supporting the enhancement of ZIKV infection in the presence of DENV immune serum, we positively enriched CD14+ monocytes from PBMCs via magnetic microbeads before performing virus inoculation. PBMCs (Fig 4A, left panel) were separated into CD14+ monocytes enriched (96.7%) population in which only 2.00% of cells were CD3+ T cells and negligible number of CD19+ B cells (Fig 4A, middle panel), and the CD14- fraction in which only a small proportion of CD14+ cells left (3.19%), whereas the proportions of T cells and B cells remained unchanged (Fig 4A, right panel). Each of the fractions was subjected to ADE assays with ZIKV (MOI: 2) in the presence of a control serum sample (Naïve-01, 1/100) or a fixed dilution of DENV immune sera that had been shown to enhance ZIKV infection (D1-E01, 1/2,500 dilution; D1-E06, 1/2,500 dilution; D3-L01, 1/100 dilution). To control for ADE infection in the absence of DENV immune serum, the cells were also infected by ZIKV at a range of MOIs (0, 0.2, 2, 20). The supernatants from each of the experimental conditions were collected and subsequently applied to Vero cells to perform a boosted infection assay as shown previously in Fig 3B. As expected, DENV immune serum samples, rather than the naïve control serum, markedly enhanced ZIKV infectivity in PBMCs (Fig 4B). Compared with the CD14- cell fraction, ZIKV infectivity was significantly greater in CD14+ monocytes enriched fraction under ADE conditions (Fig 4C). In the absence of enhancing serum, the ZIKV infection was barely detectable at an MOI of 0.2 or 2, whereas higher levels of virus infection were observed at an MOI of 20 in PBMCs and CD14+ monocytes (Fig 4B and 4C). Collectively, these results (Figs 3 and 4, Table 2) provided strong evidence that human monocytes, rather than T cells or B cells within PBMCs are the principal target cells mediating both direct and ADE infection of ZIKV.

### Human dendritic cells do not mediate ADE of ZIKV infection

**Fig 5:**
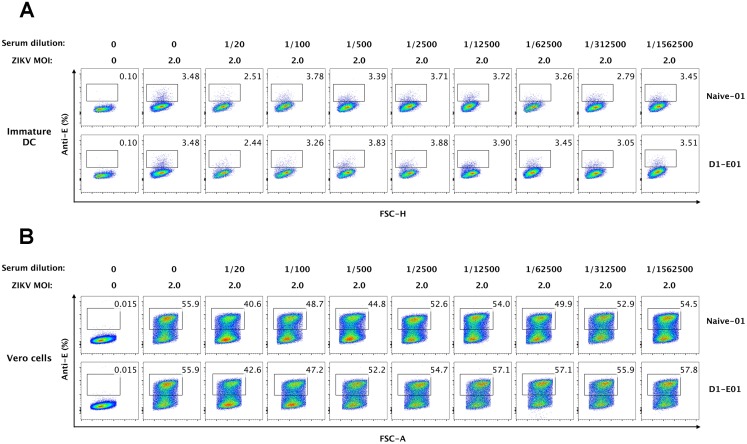
Immature DCs do not mediate ADE of ZIKV infection. (A) Immature DCs were infected with ZIKV (MOI 2) in the presence or absence of serially diluted DENV immune serum (D1-E01) or DENV-naïve serum (Naïve-01). 48 h post-infection, cells were harvested and intracellularly stained with an anti-E antibody and subjected to flow cytometry analysis. A representative infection panel of two independent experiments was presented. (B) Supernatants from ZIKV-infected immature DCs were collected and inoculated to Vero cells for 2 days. Vero cells were collected and intracelullarly stained with an anti-E antibody for flow cytometry analysis. Data shown were representative results of three independent experiments.

**Fig 6:**
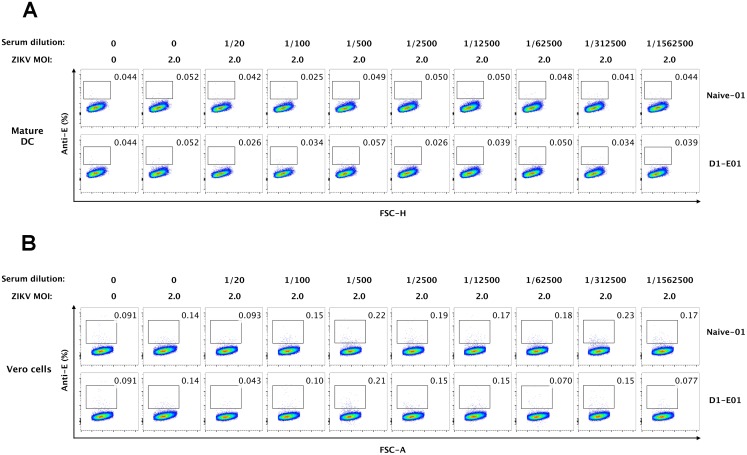
Mature DCs are not infected by ZIKV in the presence or absence of enhancing serum. (A) Mature DCs were infected by ZIKV at an MOI of 2 with or without serially diluted DENV immune serum (D1-E01) or DENV-naïve serum (Naïve-01) and cultured for 48 h. Cells were collected and stained for ZIKV-infected cells (anti-E positive) and then analyzed by flow cytometry. (B) Supernatants from ZIKV or virus/sera immune complex treated mature DCs were used to infect Vero cells for 48 h, and cells were stained for flow cytometry to identify ZIKV-infectivity. Data shown were representative results of three independent experiments.

Previous studies have demonstrated ZIKV infected DCs *in vitro* [18, 20], thus we further examined whether DCs are target cells mediating ADE of ZIKV infectivity in the presence of DENV immune sera. As expected, our results showed that HLA-DR+ CD11c+ myeloid DCs (mDCs) are a minor population in PBMCs (less than 0.30% among CD3-CD14-CD19- cell subset) (Figure A in S2 Fig). Due to the rarity of DCs in PBMCs, to gain a better understanding of the role of DCs in the enhancement of ZIKV infectivity, we generated immature and mature monocyte-derived DCs (moDCs) from healthy donors and infected them with ZIKV at an MOI of 2 in the presence or absence of serially diluted DENV immune sera (D1-E01) (Figures B and C in S2 Fig). Interestingly, immature DCs were efficiently infected by ZIKV with an infection rate of approximate 3.5%, whereas mature DCs were not permissive to ZIKV infection (Figs 5A and 6A). When DENV immune sera were applied at a wide range of serum dilutions, increased ZIKV infections were not observed both in immature DCs and mature DCs. Notably, human sera under a low dilution (1/20) seemed to exhibit some inhibition of ZIKV infection in immature DCs, possibility due to the non-specific binding of ZIKV with high concentrations of human antibodies (Fig 5A). The subsequent boosted infection assays in Vero cells with supernatants from ZIKV-inoculated DC further verified the high sensitivity of immature DCs and non-permissiveness of mature DCs to ZIKV infection. Again, results from Vero amplification assays confirmed that despite distinct susceptibilities to ZIKV infection, neither immature DCs nor mature DCs mediated ADE of ZIKV infection (Figs 5B and 6B).

### Sampling times of DENV immune sera rather than DENV serotypes may contribute to diverse ZIKV enhancing activities

**Fig 7:**
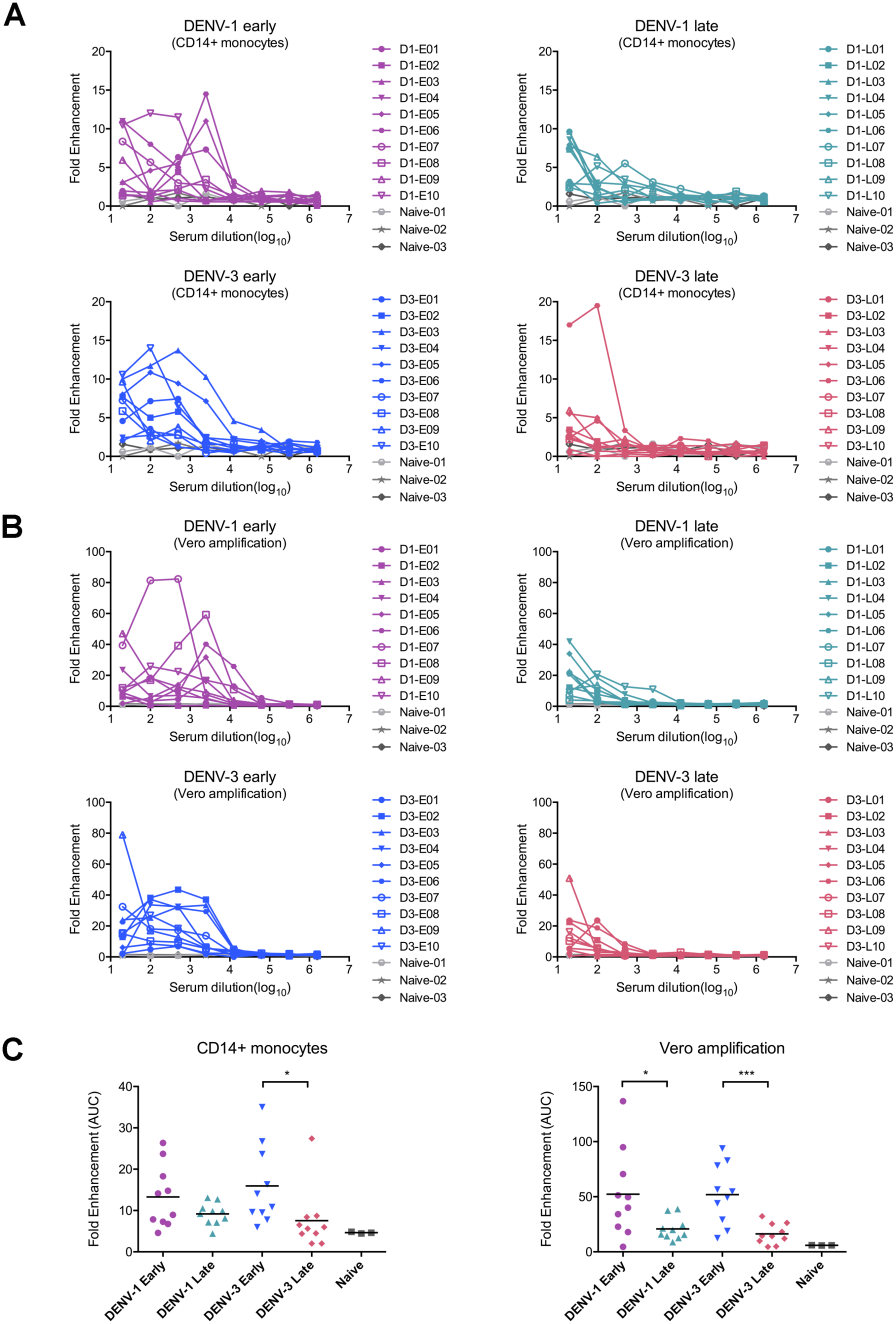
DENV immune sera from patients under different stages of convalescent phase exhibit divergent enhancing activities to ZIKV infection. All DENV immune sera and DENV-naïve sera were evaluated for ADE of ZIKV infection in PBMCs first and then by the boosted assay on Vero cells. The fold enhancement of each serum sample was presented under serial dilutions in monocytes (A) and Vero cells (B). (C) Summary of enhancing activities in monocytes and the results of boosted assay on Vero cells by calculating the area under curve (AUC) of the data from (A) and (B). The solid line represented the mean value of AUC in each group. Statistical significances were determined using unpaired two-tailed Student’s *t* test. “*” represents p<0.05, and “***” represents p<0.001.

Whether sera obtained from patients infected by different DENV serotypes influence their enhancing capacity of ZIKV infection was previously unknown. In the current study, we observed similar patterns of enhancement in ZIKV infectivity by DENV immune sera obtained from DENV-1 and DENV-3 infected individuals in our primary cell system. All the DENV-1 immune sera (n = 20) and 18/20 DENV-3 immune serum samples were able to enhance ZIKV infection over the range of serum dilution tested (1/20-1/1,562,500) in primary monocytes and further verified in subsequent Vero cell amplification assays (Fig 7A and 7B). Although two samples in DENV-3 group exhibited no appreciable ZIKV enhancing activities, there were no differences in overall potency of enhancing activities between DENV-1 and DENV-3 immune sera (DENV-1 early versus DENV-3 early, DENV-1 late versus DENV-3 late, Fig 7C).

However, when examining samples collected at different times, we observed significantly different levels of enhancing activities. It is interesting to note that the enhancement of ZIKV infection was shown over a wide range of immune sera from early sampling times (dilutions from 1/20 to 1/2,500), but only at 1/20 and 1/100 dilutions for late collection samples both for DENV-1 and DENV-3 immune groups (Fig 7A and 7B). Furthermore, statistical analyses revealed the higher level of enhancing activities in DENV-3 early group than that of DENV-3 late group in primary monocytes. Of note, despite no significant differences between DENV-1 early group and the late group in primary monocytes, upon Vero cell amplification, samples in DENV-1 early group exhibited obviously higher enhancing activity to ZIKV infection than the late group. As expected, samples from DENV-naïve individuals (n = 3) did not evoke ADE of ZIKV infection.

### Fc receptors including FcγRI (CD64), FcγRII (CD32) and FcγRIII (CD16) mediate ADE of ZIKV infection triggered by DENV immune sera

**Fig 8:**
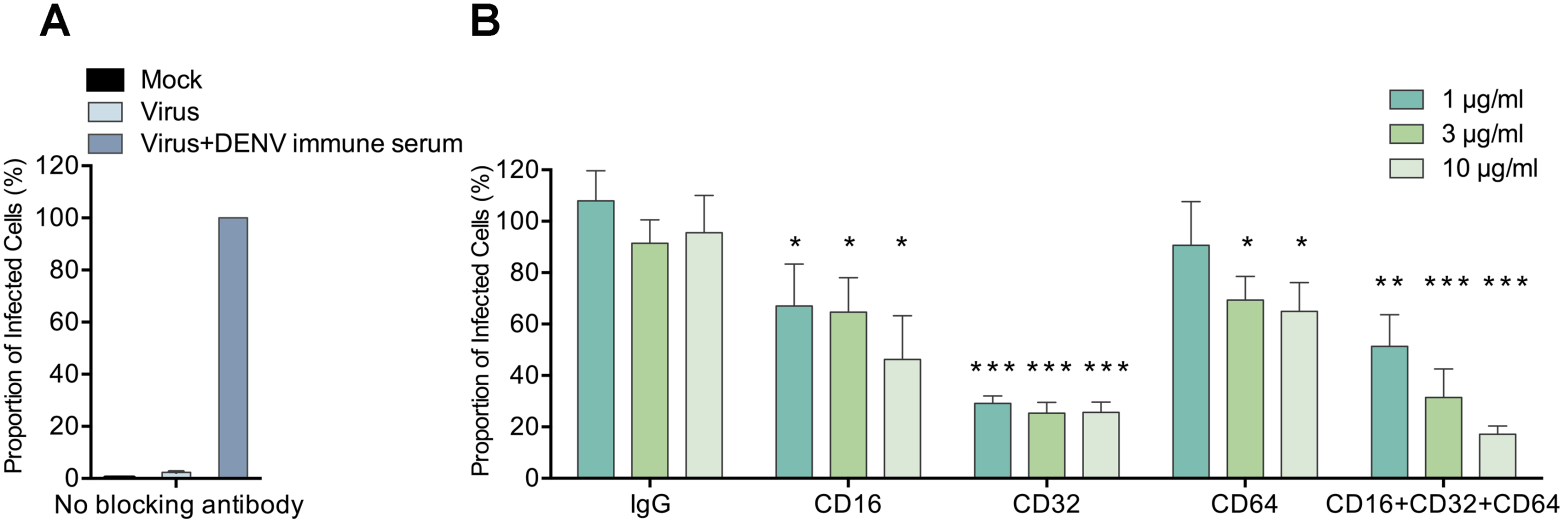
FcγRI (CD64), FcγRII (CD32) and FcγRIII (CD16) are all involved in ADE of ZIKV infection by DENV immune sera. PBMCs from healthy donors (n = 3) were treated with monoclonal antibodies (1 μg/ml, 3 μg/ml or 10 μg/ml) against each FcγR types or combined (1 μg/ml, 3 μg/ml or 10 μg/ml in total), or with IgG control antibody prior to performing the boosted ZIKV ADE assay with DENV-1 immune serum (D1-E01, 1/2,500 dilution). The percentage of anti-E positive Vero cells in the control group, where no anti-FcγR antibodies were applied at the ADE condition was defined as 100%. The mean and standard deviation of results from three independent experiments without blocking antibody treatment (A) or with blocking antibody treatment (B) were presented. Statistical significances were determined by comparison of the relative proportion of infected cells with blocking antibody treatment to that of the control IgG antibody treatment under the same antibody concentration using unpaired two-tailed Student’s *t* test and designated as “*”for p<0.05, “**” for p<0.01, and “***” for p<0.001.

Having confirmed that ADE infection of ZIKV can be reproducibly detected in primary monocytes which express all three types of Fcγ receptors, we went on to examine the relative contribution of each receptor. Freshly isolated PBMCs from three healthy donors were treated with monoclonal antibodies individually against FcγRI (CD64), FcγRII (CD32), FcγRIII (CD16), or combined together prior to performing the ZIKV ADE assay (MOI: 2) with DENV immune serum (D1-E01, 1/2,500 dilution), using isotype-matched IgG as negative controls. The cell culture supernatant from each experimental conditions was then collected and applied to Vero cells in the boosted infection assay as described in Fig 3B. To quantify the results, we set the proportion of anti-E positive cells in ADE infection without blocking antibody as 100%. Accordingly, the staining background in mock-infected group and virus-only group were 0.3% and 2.3%, respectively (Fig 8A). In anti-FcγR antibody treated groups, significantly inhibited ADE levels were observed in a dose-dependent manner for each blocking antibody or all three antibodies combined. In the IgG control group, the levels of ADE infection were not affected at any antibody concentration (Fig 8B). Anti-FcγRII (CD32) antibody presented the strongest inhibition against ADE of ZIKV infection (as much as 75% inhibition), suggesting that FcγRII may serve as the main receptor mediating ADE of ZIKV infection by DENV immune sera. In addition, anti-FcγRIII (CD16) or anti-FcRI (CD64) antibody showed significant but lower inhibition of 20%-55% dependent on the concentration of antibodies used. The combination of all three antibodies induced as much as 83% inhibition of the ADE infection of ZIKV, with a total antibody concentration of 10 μg/ml.

## Discussion

Because of the taxonomic and structural similarity between ZIKV and DENV, whether preexisting antibodies to DENV elicited during natural infection or by vaccination can facilitate ZIKV infection and exacerbate diseases due to antibody-dependent enhancement is of great concern. To better understand the serological interplay between ZIKV and DENV, we examined the direct and ADE infection of ZIKV using a primary cell experimental system that we have established in the study of DENV [32, 36]. In the current study, we have found that primary monocytes, rather than T cells or B cells were the principal target cells permissive to ZIKV infection in PBMCs, and monocytes could mediate enhanced ZIKV infectivity in the presence of diluted DENV-1 or DENV-3 immune human sera. Additionally, immature DCs and mature DCs were not able to mediate ADE of ZIKV infection in the presence of DENV immune sera. Furthermore, antibody blocking of FcγRI (CD64), FcγRII (CD32) and FcγRIII (CD16) individually, or combined, markedly decreased the magnitude of ADE of ZIKV infectivity in primary monocytes. To our knowledge, this is the first detailed characterization of ADE infection of ZIKV in a primary cell culture system, and specific Fcγ receptors involved in the ADE of ZIKV infection.

Our results expand the existing knowledge on ADE of ZIKV infection. Several previous studies provided evidences indicating enhancing activities of preexisting DENV immunity on ZIKV infection. Serum samples from DENV-infected patients had been shown to promote enhancement of ZIKV infection in K562 and U937 cell lines [24, 25, 27, 37, 38]. In addition, human monoclonal antibodies against DENV showed various degrees of enhancing activities for ZIKV infection in FcγR-bearing cell lines [24–26, 39]. Additionally, polyclonal sera from ZIKV-exposed mice and human monoclonal antibodies isolated from ZIKV-infected subjects displayed ADE of DENV infections [26, 40]. However, whether the same holds true in primary human cells was not known. Our results demonstrating DENV immune sera can mediate ADE infection of ZIKV are generally consistent with some previously published data using cell lines [24, 25, 27, 37, 38]. Importantly, we extended the previous observations into human primary PBMCs, an experimental condition that mimics more closely the *in vivo* circumstance, where a variety of cell types may confront antibody-ZIKV immune complexes in an identical milieu and contribute to increased viral infectivity. To date, this observation is not yet supported by non-human primate experiments or epidemiological evidence that DENV serostatus correlates with exacerbated ZIKV outcomes in humans [41, 42], prospective epidemiology studies designed to examine this specific question are further warranted.

In previous studies, FcγR-bearing cell lines K562, U937 and THP-1 have been utilized to determine immune enhancement of ZIKV infectivity in the presence of DENV immune serum or DENV-specific monoclonal antibodies. Compared with the primary cell system, however, these cell lines have distinct insufficiencies for ADE study. K562 is a human erythroleukemia cell line, whereas U937 (human pro-monocytic leukemia cell line) and THP-1 (human monocytic leukemia cell line) are undifferentiated cells that do not have the same biological properties of primary human monocytes which express high levels of both FcγRII (CD32) and FcγRI (CD64), as well as low levels of FcγRIII (CD16). In contrast, the above mentioned cell lines express intermediate to low levels of FcγRI (CD64) and no FcγRIII (CD16) on the surface, despite similar high levels of FcγRII (CD32), notably, K562 cells only express FcγRIIa [36, 43]. Other than differences in Fc receptor expression, K562 cells do not have normal type I interferon responses; U937 and THP-1 cells are immature cells that do not recapitulate the function of mature monocytes, and thus these cells are not suitable for in depth investigation of intrinsic mechanisms of ADE [43, 44]. During ADE of DENV infection in primary monocytes, both FcγRI (CD64) and FcγRII (CD32) were involved [32, 35, 45]. Due to its low affinity, FcγRIII (CD16) was not considered to be as important as FcγRI (CD64) and FcγRII (CD32) in mediating ADE of DENV infection. The same may not be true for Zika virus. Some recent studies indicated CD14+ CD16+ monocytes to be the main target of ZIKV infection in PBMCs, and ZIKV infection induced a phenotypic shift within monocytes toward increased CD16 expression, suggesting a possible role of FcγRIII (CD16) during ADE of ZIKV infection [19, 20]. Indeed, our results confirmed that all three types of FcγRs including FcγRIII (CD16) contributed to ADE of ZIKV infection. Only by using primary cells, this mechanism could be revealed. Studies using cell lines, however, often do not allow making the same observations. Thus, it would be reasonable to suggest that preliminary conclusions made in cell lines, with respect to ADE studies, should be further validated in a primary cell system.

Compared to DENV that infected an average 1%-5% of primary monocytes at MOI of 1–5 in the absence of enhancing antibodies [32, 35], primary monocytes exhibited less permissiveness to ZIKV in our study, with a 0.040% infection rate on average at an MOI of 2 (Fig 3A and Table 2). A recent study using whole blood cells from healthy donors revealed significantly higher viral infectivity in CD14+ monocytes with African strain (MR766) than those infected with the Asian strain (H/PF/2013) [19]. Meanwhile, DENV was shown to infect markedly more PBMCs in DENV-infected patients than ZIKV in ZIKV-infected subjects [20]. It remains to be determined whether the limited ZIKV permissiveness observed in our study was restricted to the specific Asian ZIKV strain used here (SZ-WIV01), or a fundamental difference between DENV and ZIKV.

Other than CD14+ monocytes, we sought to further identify the role of DCs during ADE of ZIKV infection triggered by DENV immune sera. Consistent with previously published reports on DENV, we found that immature DCs were highly sensitive to ZIKV, but did not mediate ADE, possibly due to the high expression level of DC-SIGN on the surface of immature DCs [46]. Different from that reported for DENV, in our study, mature DCs were not infected by ZIKV, regardless of the presence or absence of enhancing sera. A possible explanation for these divergent observations may be that ZIKV or virus/antibody immune complex inoculation onto mature DCs might have led to the activation of innate immune signaling pathways and triggered the production of type I and type II interferon and other related antiviral factors that limit the infection of ZIKV. How ZIKV influences immune signaling pathways of DCs and mechanisms underlying different sensitivities between immature DCs and mature DCs require further investigation.

The E protein of ZIKV (SPH2015) shares similar sequence identities with four dengue serotypes, showing 56.0% (DENV-1 16007), 53.1% (DENV-2 16681), 56.9% (DENV-3 16561) and 54.7% (DENV-4 1036) homology, respectively. To elucidate the serological cross-reactivity between ZIKV and DENV, we utilized a panel of serum samples obtained from patients during convalescent phase of either DENV-1 or DENV-3 infection. According to our results, these two groups of sera exhibited similar patterns of binding, neutralization and ADE to ZIKV. Whether this observation holds true for all four DENV serotypes and potentially diverse genotypes warrants further studies. Samples collected at different times post-infection exhibited different potency of ADE, showing that early DENV immune sera presented higher enhancing activities than late sera, which is in accordance with the results that early sera had higher binding capacity toward ZIKV than late sera (Fig 2B). Based on this observation, it is reasonable to speculate that the titers of both serotype-specific and ZIKV cross-reactive antibodies in the DENV immune sera may decline over time, and thus directly influenced their enhancing capacity of ZIKV infection. A follow-up study using longitudinal serial DENV immune serum samples in larger sample numbers would help to verify this hypothesis.

In summary, we have demonstrated that primary monocytes, not B cells, T cells or dendritic cells in peripheral blood are the principal target cells mediating enhanced ZIKV infectivity in the presence of DENV immune sera. Time post infection of DENV, instead of dengue serotype specificity, appears to influence the ADE activities of dengue immune sera on ZIKV infection. Moreover, all three types of Fcγ receptors play important roles in ADE infection of ZIKV. These findings highlight the importance of further investigation on ZIKV infection in the presence of preexisting antibody responses to other flaviviruses. Furthermore, our results as well as published papers from others [24–27, 37, 38], unequivocally confirmed that Zika immune sera can enhance DENV infection, and dengue immune sera can enhance ZIKV infection in cell lines, primary cells, and small animals. Thus the testing of ADE activity should be an integral part of immunological tests during dengue vaccine trials, as well as Zika vaccine studies. Additionally, it is imperative to examine or monitor signs of ADE in the preclinical and clinical testing of new dengue and Zika vaccines.

## Supporting information

S1 Fig Gating strategies of monocyte, T cell and B cell subsets within PBMCs. (TIF)

S2 Fig Gating strategies and phenotype analyses of dendritic cells. (A) Gating strategies of myeloid dendritic cells (mDCs) within PBMCs. mDCs (HLA-DR+ CD11c+ CD3- CD14- CD19-) comprise a small proportion of total PBMCs. (B) Monocyte-derived dendritic cells were generated from CD14+ monocytes in the presence of IL-4 and GM-CSF and the subsequent LPS stimulation. Gating strategies of monocyte-derived immature DCs and mature DCs were presented. (C) Phenotypes of immature DCs and mature DCs were characterized by flow cytometry.
(TIF)
