# Supplementary figures and images for: Dengue immune sera enhance Zika virus infection in human peripheral blood monocytes through Fc gamma receptors
Source: PLoS One. 2018 Jul 25;13(7):e0200478. doi: 10.1371/journal.pone.0200478 (PMC6059439; doi:10.1371/journal.pone.0200478)

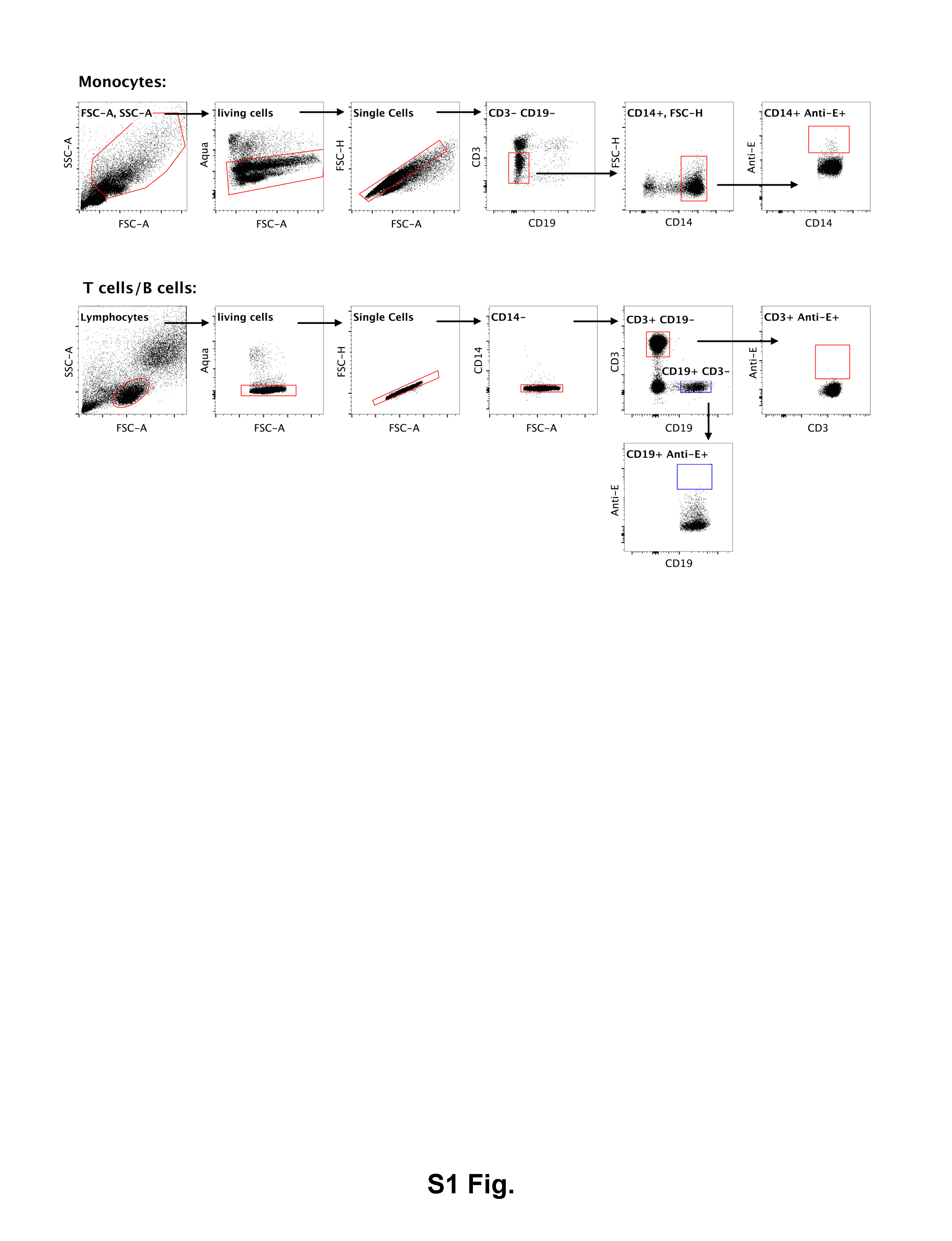

Supplement: S1 Fig — (TIF) [file pone.0200478.s001.tif]

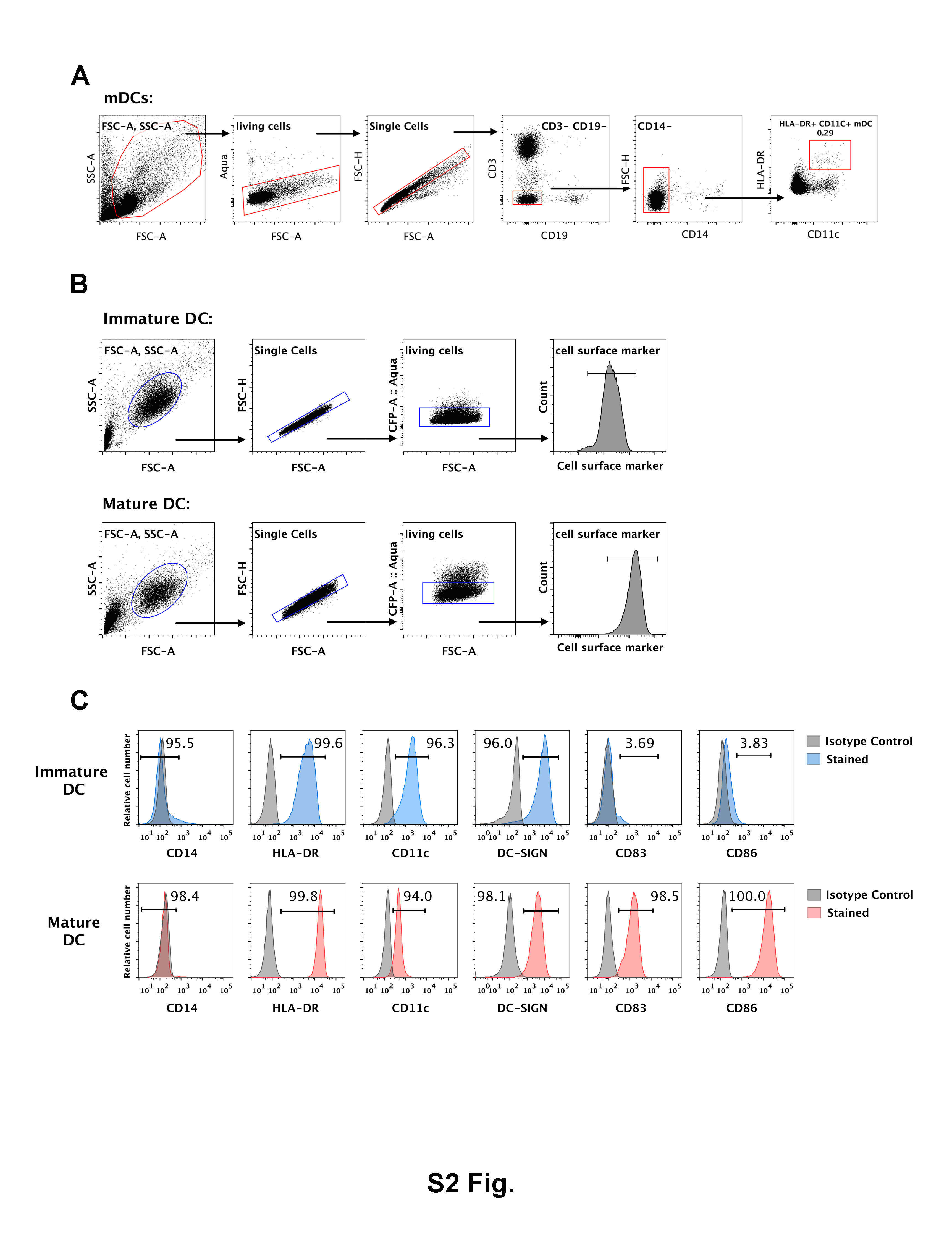

Supplement: S2 Fig — (A) Gating strategies of myeloid dendritic cells (mDCs) within PBMCs. mDCs (HLA-DR+ CD11c+ CD3- CD14- CD19-) comprise a small proportion of total PBMCs. (B) Monocyte-derived dendritic cells were generated from CD14+ monocytes in the presence of IL-4 and GM-CSF and the subsequent LPS stimulation. Gating strategies of monocyte-derived immature DCs and mature DCs were presented. (C) Phenotypes of immature DCs and mature DCs were characterized by flow cytometry. (TIF) [file pone.0200478.s002.tif]
